# Special Issue: Layered Double Hydroxides (LDH) and LDH-Based Hybrid Composites

**DOI:** 10.3390/ma14102582

**Published:** 2021-05-16

**Authors:** Jakub Matusik

**Affiliations:** Department of Mineralogy, Petrography and Geochemistry, Faculty of Geology, Geophysics and Environmental Protection, AGH University of Science and Technology, al. Mickiewicza 30, 30-059 Krakow, Poland; jmatusik@agh.edu.pl

## 1. Introduction

LDHs are a class of two-dimensional layered anionic structures with unique properties [1]. They are composed of positively charged brucite-like layers and charge balancing hydrated anions located in the interlayer. The features of LDH phases include the following: ease of synthesis, controllable and flexible chemical composition, and a relatively large surface area. These contribute to their potential applications in adsorption-based processes, catalysis, electrochemistry, polymer chemistry, biomedicine, and wastewater treatment [2,3]. In recent years, LDH materials have been extensively studied as components of hybrid materials [4,5]. It is possible and desirable for the support to play a synergistic role in the tested system, e.g., it can induce dual adsorption properties or increase the composite stability at a low pH. Such an approach can also reduce the production costs of the materials, provided that the active LDH component is in a sufficient amount for the desired application.

## 2. Comment on the Published Articles

The research articles published in this Special Issue cover different aspects connected to LDH materials. In particular, these include: synthesis and characterization by various experimental approaches, factors affecting exfoliation efficiency, the extent of memory effect in LDH composites, adsorption properties and drug delivery applications as well as their role in environmental chemistry. The published articles are shortly characterized below.

LDH are rare in the environment; however, their importance in biogeochemical cycles was revealed by Doggaz et al. [6]. Green rusts are Fe(II)-Fe(III) LDH, which can interact with Mn(III)-Mn(IV) birnessite. The detailed study by XRD, XPS, and Mössbauer spectroscopy showed the possibility of Fe(II) oxidation by birnessite, which leads to ferromanganese spinels.

Nanohybrids of Zn/Al LDH with graphene oxide are an example of composites showing dual adsorption properties. This means that such materials can simultaneously remove cationic and anionic pollutants from wastewaters. Their high efficiency was reported in the study by Saber et al. [7].

The exfoliation ability of LDH is of high importance for many applications including polymer nanocomposite preparation, where the homogenous dispersion of the filler is required. The study of Karthikeyan et al. [8] enabled the identification of factors responsible for the efficient exfoliation of nitrate intercalated Mg/Al LDH.

Mycotoxins in feed and food are highly toxic and pose a serious danger even at very low concentrations. Bentonites in animal diets can reduce toxin bioavailability. However, some mycotoxins such as fumonisin B1 form anionic species, which excludes the use of negatively charged clays. The research by Matusik and Deng [9] confirmed a high affinity and selectivity of LDH structures towards anionic forms of fumonisin B1.

Memory effect is an unique property of LDH. It leads to the reconstruction of layered structures after its calcination under appropriate conditions. Bezerra et al. [10] investigated the memory effect phenomena by in situ XRD for Mg/Al-LDH, as well as for its composite with zeolite.

The research by Maziarz et al. [11] showed the advantages of Mg/Al LDH use together with Ca(OH)_2_ as a mixture for sulfate removal from AMD water (acid mine drainage). The applied methodology resulted in a significant reduction of sludge volume in comparison to the experiments where only Ca(OH)_2_ was used. Additionally, the settling time was significantly enhanced, as indicated by lower turbidity in the treated wastewater.

A composite material consisting of Mg/Al LDH and halloysite was prepared in the study of Matusik et al. [12]. Its adsorption effciency in reactions with aqueous As(V) and Cr(VI) was investigated. The use of materials composed of two different minerals is promising due to the reduction of costs, as well as the prevention of adsorbent swelling. This allows for the possibility of LDH-based adsorbents’ use in dynamic adsorption column systems.

Drug delivery is a hot topic in materials chemistry dealing with the application of different mineral-based supports capable of transporting and releasing drugs. Lerner et al. [13] synthesized a novel bio-hybrid drug delivery system that was based on nitrate intercalated Mg/Al LDH. This allows for the possibility of designing other systems for the sustained release of anionic and neutral drugs.

The use of additional molecules in the synthesis routes of LDH helps to control their properties. The study by Michalik et al. [14] showed the effects of using starch as a structure-controlling biotemplate. This approach enabled the preparation of nanocrystalline Mg/Al LDH with a very fine (<50 nm) particle size.

The work by Alcântara et al. [15] showed a basic study on the preparation of biohybrids based on the corn protein zein and LDH for the first time. The obtained biohybrid materials could have potential interest for biomedicine, biosensing, materials for electronic devices, catalysis, and photocatalysis.

## 3. Summary

The published research articles of this Special Issue indicate the importance of LDH materials in selected applications. As shown in the published reports, LDH materials are very diverse, especially in terms of their chemical composition and surface properties. Moreover, these materials can be easily modified, as well as used as the components of composites. The selection of appropriate components (e.g., aluminosilicates, oxides), as well as chemicals for modification, allows for the adjustment of their properties for applications in industry and environmental protection. The studies have also shown that research on LDH is highly interdisciplinary, and involves researchers from scientific centers representing various disciplines undertaken by chemists, geologists, physicists, and biologists. Interest in the use of synthetic layered minerals is constantly growing for new technologies. Apart from their use as adsorbents in purification and catalysis, the potential of LDH materials increases in applications connected to medicine, the preparation of polymer nanocomposites, or intelligent materials sensitive to radiation.

## References

[1] Forano C., Costantino U., Prévot V., Gueho C.T., Bergaya F., Lagaly G. (2013). Layered Double Hydroxides (LDH). Developments in Clay Science.

[2] Mishra G., Dash B., Pandey S. (2018). Layered double hydroxides: A brief review from fundamentals to application as evolving biomaterials. Appl. Clay Sci..

[3] Zubair M., Daud M., McKay G., Shehzad F., Al-Harthi M.A. (2017). Recent progress in layered double hydroxides (LDH)-containing hybrids as adsorbents for water remediation. Appl. Clay Sci..

[4] Gómez-Avilés A., Aranda P., Ruiz-Hitzky E. (2016). Layered double hydroxide/sepiolite heterostructured materials. Appl. Clay Sci..

[5] Gan F., Luo Y., Hang X., Zhao H. (2016). Heterocoagulated clay-derived adsorbents for phosphate decontamination from aqueous solution. J. Environ. Manage..

[6] Doggaz A., Coustel R., Durand P., Humbert F., Ruby C. (2020). Birnessite: A New Oxidant for Green Rust Formation. Materials.

[7] Saber O., Asiri S.M., Ezzeldin M.F., El-Azab W.I.M., Abu-Abdeen M. (2020). Designing Dual-Effect Nanohybrids for Removing Heavy Metals and Different Kinds of Anions from the Natural Water. Materials.

[8] Karthikeyan J., Fjellvag H., Bundli S., Sjastad A.O. (2021). Efficient Exfoliation of Layered Double Hydroxides; Effect of Cationic Ratio, Hydration State, Anions and Their Orientations. Materials.

[9] Matusik J., Deng Y. (2020). Fumonisin B1 Interaction with Mg-Al and Mg-Fe Layered Double Hydroxides: Removal Efficiency and Mechanisms. Materials.

[10] Bezerra B.G.P., Bieseki L., Mello M.I.S., Silva D.R.D., Rodella C.B., Pergher S. (2021). Memory Effect on a LDH/zeolite A Composite: An XRD In Situ Study. Materials.

[11] Maziarz P., Matusik J., Leiviska T. (2019). Mg/Al LDH Enhances Sulfate removal and Clarification of AMD Wastewater in Precipitation Processes. Materials.

[12] Matusik J., Hyla J., Maziarz P., Rybka K., Leiviska T. (2019). Performance of Halloysite-Mg/Al LDH Materials for Aqueous As(V) and Cr(VI) Removal. Materials.

[13] Lerner D.A., Begu S., Aubert-Pouessel A., Polexe R., Devoisselle J.M., Azais T., Tichit D. (2020). Synthesis and Properties of New Multilayer Chitosan@layered Double Hydroxide/Drug Loaded Phospholipid Bilayer Nanocomposite Bio-Hybrids. Materials.

[14] Michalik A., Napruszewska B.D., Walczyk A., Krysciak-Czerwenka J., Duraczynska D., Serwicka E.M. (2020). Synthesis of Nanocrystalline Mg-Al Hydrotalcites in the Presence of Starch-the Effect on Structure and Composition. Materials.

[15] Alcantara A.C.S., Darder M., Aranda P., Ruiz-Hitzky E. (2020). Zein-layered hydroxide biohybrids: strategies of synthesis and characterization. Materials.

